# The smell of success: Reproductive success related to rub behavior in brown bears

**DOI:** 10.1371/journal.pone.0247964

**Published:** 2021-03-03

**Authors:** Andrea T. Morehouse, Anne E. Loosen, Tabitha A. Graves, Mark S. Boyce

**Affiliations:** 1 Department of Biological Sciences, University of Alberta, Edmonton, Alberta, Canada; 2 Winisk Research and Consulting, Bellevue, Alberta, Canada; 3 Faculty of Applied Ecology, Agricultural Sciences and Biotechnology, Inland Norway University of Applied Sciences, Koppang, Norway; 4 U.S. Geological Survey, Northern Rocky Mountain Science Center, West Glacier, Montana, United States of America; Universita degli Studi di Sassari, ITALY

## Abstract

Several species of bears are known to rub deliberately against trees and other objects, but little is known about why bears rub. Patterns in rubbing behavior of male and female brown bears (*Ursus arctos*) suggest that scent marking via rubbing functions to communicate among potential mates or competitors. Using DNA from bear hairs collected from rub objects in southwestern Alberta from 2011–2014 and existing DNA datasets from Montana and southeastern British Columbia, we determined sex and individual identity of each bear detected. Using these data, we completed a parentage analysis. From the parentage analysis and detection data, we determined the number of offspring, mates, unique rub objects where an individual was detected, and sampling occasions during which an individual was detected for each brown bear identified through our sampling methods. Using a Poisson regression, we found a positive relationship between bear rubbing behavior and reproductive success; both male and female bears with a greater number of mates and a greater number of offspring were detected at more rub objects and during more occasions. Our results suggest a fitness component to bear rubbing, indicate that rubbing is adaptive, and provide insight into a poorly understood behaviour.

## Introduction

Chemical communication is widespread in mammals and can relay information about sex, reproductive state, territory, individual identity, and dominance status [1–3]. Many species have scent glands specifically for the purpose of transmitting chemical information [2, 4]. Further, successful communication can contribute to an animal’s fitness [5], and past research has found fitness benefits to marking behavior. Communicating dominance and competitive abilities via olfactory signals can increase mating opportunities, which in turn can increase reproductive success [6–9]. For example, female laboratory mice prefer males who scent mark more frequently [7, 8]. In addition, Rothman and Mech [10] found that scent-marking was important to courtship for newly paired wolves (*Canis lupus*) as well as reproductive synchrony in established pairs, both of which are important for maximizing reproductive success.

For brown bears (*Ursus arctos*), rubbing is a common behaviour and is widely believed to represent some form of communication [11, 12]. Brown bears use a variety of marking postures but standing upright on their hind legs and rubbing their back against a surface is most common [13]. Bears rub on a variety of objects including trees, power poles, and fence posts [14, 15]. As a result of rubbing, rub objects typically develop distinguishing characteristics such as a smooth or discolored rub surface, presence of pedal marks (i.e., path worn by bears to rub object), or presence of bear hair [16–18]. These characteristics make them easily identifiable in the field. While several studies have described rubbing behaviour [e.g., 11–13, 19, 20], comparatively little research focuses on the reasons behind bear rubbing behavior.

There are currently three primary hypotheses regarding why brown bears rub, though they are not mutually exclusive. The first, and perhaps most simplistic, is that rubbing has nothing to do with communication and could simply be a way to remove hair–particularly during the spring-summer shedding period [21, 22]. The second hypothesis is that bears rub to communicate superior competitive ability (i.e., dominance) [11, 20]. Third, rubbing might function to signal for mates during the breeding season [23]. Regardless of the mechanism (i.e., mate signaling or dominance communication), if scent marking confers fitness benefits, we might expect a relationship between bear rubbing behavior and reproductive success. We expand on these hypotheses in the following paragraphs.

Brown bears might rub to remove hair, particularly during molting seasons [21, 22]. The timing of the molt depends on the bear’s nutritional status because the energy and protein demands of hair growth compete with other physiological processes [24, C. T. Robbins, personal communication]. Molt can begin in May or can be delayed into late summer or fall depending on the bear’s nutritional intake relative to all other demands. For example, young bears prioritize growth while females with young prioritize lactation over hair growth [24, C. T. Robbins, personal communication]. Lactating females also might reduce movement to protect their cubs and this can limit their access to the highest quality foods. Thus, molting in adult, lactating females may be delayed relative to when it occurs in adult males [C. T. Robbins, personal communication]. In the context of rubbing behavior, we would expect females, particularly those with young, to start rubbing later in the year if the hair removal hypothesis was supported.

Alternatively, the dominance or mate signaling hypotheses imply that rubbing has a communication component. Brown bears are wide-ranging, solitary, and have overlapping home ranges [25–27]. Thus, it seems reasonable that they might use some form of chemical signaling to communicate with conspecifics. Brown bears possess both anal and pedal scent glands, and their secretions are thought to communicate information related to the sex of the animal [17, 28]. These scent glands may be an important component to bear rubbing behavior. For example, sitting, stomping and sniffing behavior as well as urination are common at bear rub objects [11, 29, 30, K. Kendall, personal communication], allowing for the deposition of chemical compounds.

Dominance hierarchies exist in many mammals, including brown bears, and rubbing might be one way by which bears can communicate their dominance [11, 20]. Older, larger, more-aggressive male bears typically outcompete less-dominant males in intrasexual competition for access to females during the mating season [31–33]. Similarly, more-dominant individuals often outcompete less-dominant individuals for access to food and habitat resources, which can in turn affect fitness [25, 31, 32, 34, 35]. Communicating dominance through olfactory signals can increase mating opportunities through the defence of territories, deterrence of competitors, and advertisement of competitive abilities, which might be attractive to females [6, 7, 36].

In addition to removing hair or communicating dominance, rubbing also might allow brown bears to signal for mates during the breeding season [23]. Although relatively little is known about mate choice for brown bears, both male-male competition and female choice in brown bears have been documented [25, 32, 35]. Like many other mammalian species, female brown bears are the choosier sex because they typically invest more into reproduction [34, 37, 38], and females rely on visual, acoustic, and/or olfactory signals to inform their mate selection [34, 39]. Scent marking, for example, can convey information about an individual’s condition and genetic quality [39–41]. There is increasing evidence that odours communicate genetic information that might increase fitness, including information about relatedness and nepotism [41–43]. Further, females appear to prefer genetically dissimilar males [7]. Thus, there is an interaction between good-genes indicator traits and traits signaling genetic compatibility, although this relationship is not yet well understood [44, 45].

If the most odoriferous males are more successful in securing females and siring offspring that inherit their scent glands, then olfactory cues and the development of scent glands are understandable in the context of sexual selection [34]. Thus, regardless of the mechanism (i.e., mate signaling or dominance communication), if rubbing is sexually selected, rubbing and reproductive success should be positively related for male bears. However, both male and female brown bears rub. Although scent marking is often more commonly associated with males, mammalian females also scent mark, possibly to indicate their receptiveness during the mating season, to solicit male scent marks to test potential mate quality, or to mark their home range [46–48].

Rubbing also might confer fitness benefits to rubbing female bears. Bears are polygamous breeders, and females often mate with more than one male [35, 49]. Multiple mating might be a female strategy to confuse paternity to reduce the potential of sexually selected infanticide (SSI), whereby males kill non-offspring cubs to bring the female intro estrous for mating [35, 49]. Thus, if multiple mating reduces SSI by confusing paternity and females rub to attract multiple mates, we might expect a relationship between female reproductive success and rubbing. Fitness is dependent on an individual’s ability to successfully reproduce, and reproductive success is initially dependent on the ability to secure mating opportunities.

Our objective was to evaluate the relationship between brown bear reproductive success and rubbing behavior. Specifically, we evaluated the prediction that bears that rub more frequently will have a greater number of mates and more offspring. If rubbing is primarily for hair removal, we did not expect to see a relationship between rubbing behaviour and the number of offspring. However, if rubbing is related to communication, either by relaying dominance information or mate signaling, we predicted that we would detect a positive relationship between an individual’s reproductive success and the number of rub objects at which and occasions during which they were detected. Further, we expected to see this positive relationship between reproductive success and rubbing behavior for both male and female brown bears.

## Study area

**Fig 1:**
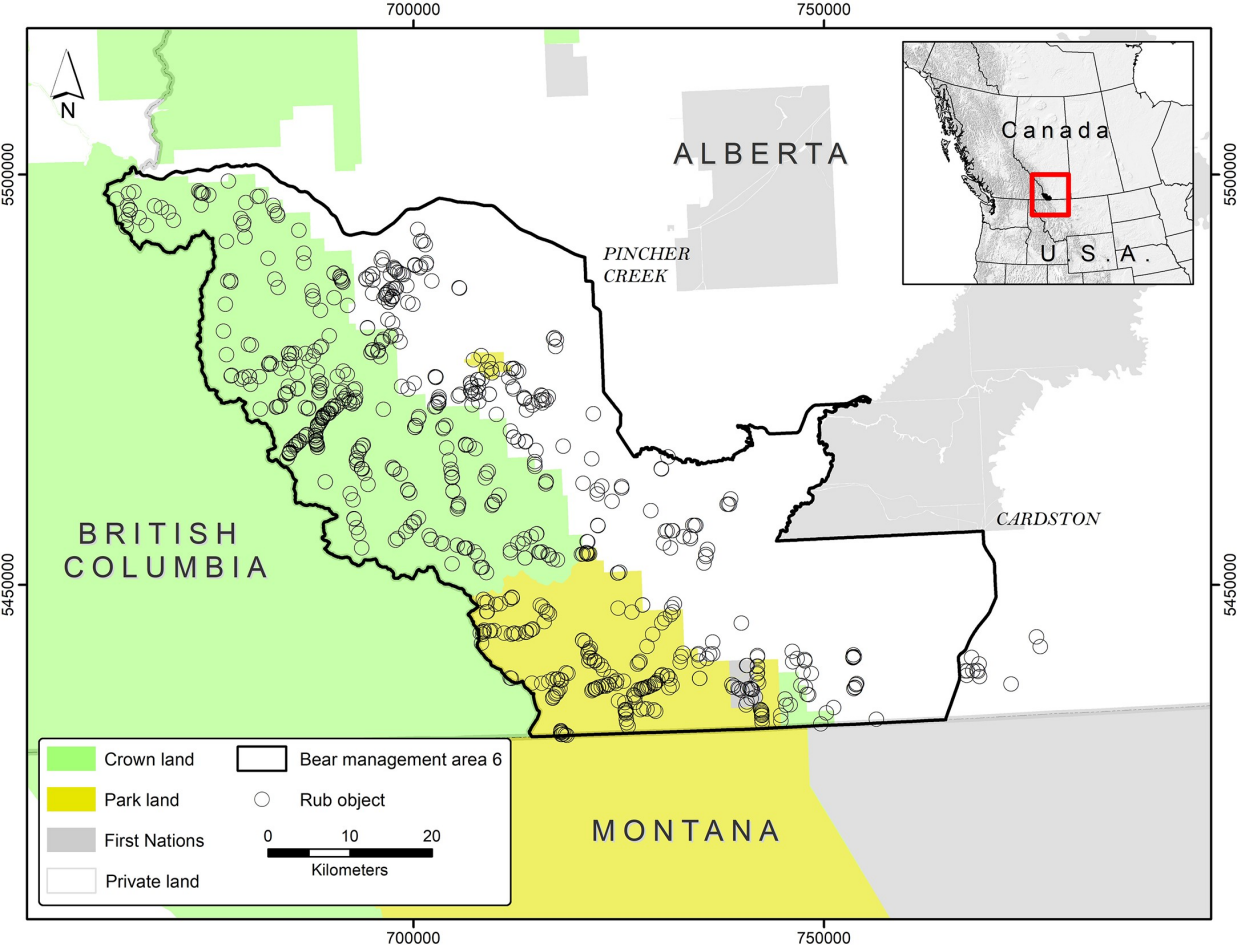
Study area. Map of the study area in southwestern Alberta, Canada.

Our study area was in southwestern Alberta, Canada, where the northern boundary was trans-Canada Highway 3, the western boundary was the British Columbia border, the southern boundary was Montana, USA, and the eastern boundary encompassed most of the eastern extent of brown bear range (Fig 1). In our study area, the mountains transition abruptly to prairie and agricultural areas. Strong winds (>100 km/hr) were common, and the climate was characterized by cold winters and warm, dry summers. The study area was a mix of mountainous, forested public lands (48%) under the jurisdiction of the provincial and federal (Waterton Lakes National Park) governments. Oil and gas development as well as forestry and timber harvest were present on public lands. The remaining 52% of the study area was privately owned land, where the predominant land use was agriculture and included both livestock and crop production.

All four native large carnivores were present; brown bears, black bears (*U*. *americanus*), cougars (*Puma concolor*), and wolves. Approximately 67.4 (95% CI 50.0–91.1) resident brown bears had home range centers in our study area, and 172 brown bears used the study area at some time each year [15].

## Materials and methods

From 2011 through 2014, we identified 899 unique rub objects throughout the study area [15]. For this paper, we included only rub objects, defined as trees, power poles, and fenceposts (*n* = 822); we excluded stretches of barbed wire fence that bears passed through. Each rub object was uniquely numbered, and we attached 4-pronged barbed wire to the rub object to facilitate hair collection and provide discrete sampling units. We determined individual identity from DNA extracted from hair follicles [50, 51]. We included data from 4 years of sampling (2011–2014). The first two years of the project were primarily set-up years and rub objects were visited less frequently than the last two years (2011: 2 sampling occasions on Crown and Park lands only, 2012: 7 sampling occasions on Crown and Park lands and 2 sampling occasions on private lands). During 2013 and 2014 all rub objects were visited 8 times from late May through early November, resulting in 7 sampling occasions. Each sampling occasion for all years was 3 weeks. We also collected hair samples opportunistically from private agricultural lands in the eastern portion of our study area. After each hair collection, we passed a flame over each barb to prevent contamination in the next sampling cycle. Further details of the sampling methods can be found in [15] and [52]. We genotyped 213 individual brown bears (118 males, 95 females) at 24 microsatellite loci, plus the amologenin sex marker [52]. All field methods were completed in accordance with the Canadian Council on Animal Care guidelines and approved by the University of Alberta Bio Sciences Animal Care and Use Committee (Protocol # AUP00000008). Field permits were granted by Alberta Environment and Parks, and Parks Canada.

In a parentage analysis, one of the primary causes of incorrect parent assignment is incomplete sampling of candidate parents [53–55]. Brown bears in southwestern Alberta are a small part of a larger Rocky Mountain sub-population of brown bears that extends into British Columbia and Montana, USA [27]. Thus, we obtained data from previous non-invasive genetic sampling projects in Alberta [56], British Columbia [57], and Montana [14, 58, 59] and included these data in our parentage analysis to increase our likelihood of identifying complete triads (mother, father, offspring) [52]. We used 2,043 individual genotypes (977 males, 1072 females) in our parentage analysis. There were 6 cases where sex was unknown, and we analyzed those bears as both potential mothers and potential fathers [52].

We used program COLONY [60] to assign parentage. COLONY uses a full pedigree approach to simultaneously assign parentage and sibship [60]. For the parentage analysis, we specified the following parameters: polygamous males and females, long run length (~1.9 billion iterations), full-likelihood analysis, medium-likelihood precision, initial proportion of parents in the dataset at 0.4 for each sex, and genotypic error of 0.001 [58, 59]. We used known ages (age determined by cementum annuli from extracted teeth of handled bears) to exclude potential parents if they were not at least 2 years older than a potential offspring. Further details of our parentage analysis methods can be found in [52].

While we used genetic data from the entire Rocky Mountain sub-population to ensure our parentage analysis was robust, we assessed the influence of rub behavior on reproductive success only with brown bears that were detected at rub objects in our southwestern Alberta study area (*n* = 55 for females, *n* = 92 for males) because that was our focal area for intensive rub object sampling. For each Alberta bear, we determined the number of offspring, the number of mates, the number of unique rub objects where an individual was detected, and the number of sampling occasions (i.e., 3-week sampling period) during which an individual was detected. We considered all data cumulatively across all 4 years.

Using Poisson regression, we first evaluated how the number of mates varied as a function of the number of rub objects at which and occasions during which a bear was detected. Second, we assessed how the number of offspring varied as a function of the number of rub objects at which and occasions during which a bear was detected. We standardized covariates (mean = 0, SD = 1) and examined each explanatory variable independently (i.e., two models for each response variable) because they had high correlations (Pearson’s correlation coefficient r = 0.83 for females, r = 0.93 for males) with each other. We considered the relationship significant if the confidence intervals of the estimate did not overlap zero at the α = 0.05 level.

Next, because younger bears will have fewer offspring and mates than older bears and this could influence the relationships we were testing, we calculated a relative age covariate for bears known to have successfully reproduced to help control for the potential that age alone is driving the patterns in our results (*n* = 19 for females, *n* = 27 for males). We determined if a bear was a “parent” (all bears in reduced dataset were parents), “grandbear,” great “grandbear,” or great great “grandbear” for individuals with known offspring and assigned each category a numerical value. We used these generations as indices for relative age because no age data were available for most bears. Again, we analyzed data using Poisson regression using only individuals known to have reproduced and included relative age as an additional scaled covariate in each model. We refer to this dataset as the ‘reduced data.’ Analyses were completed for each sex separately in the statistical software R (R Version 3.6.2, https://cran.rproject.org). We calculated exponential effect sizes for each covariate [61] to compare consistency of the reduced data analysis with the full dataset.

## Results

**Fig 2:**
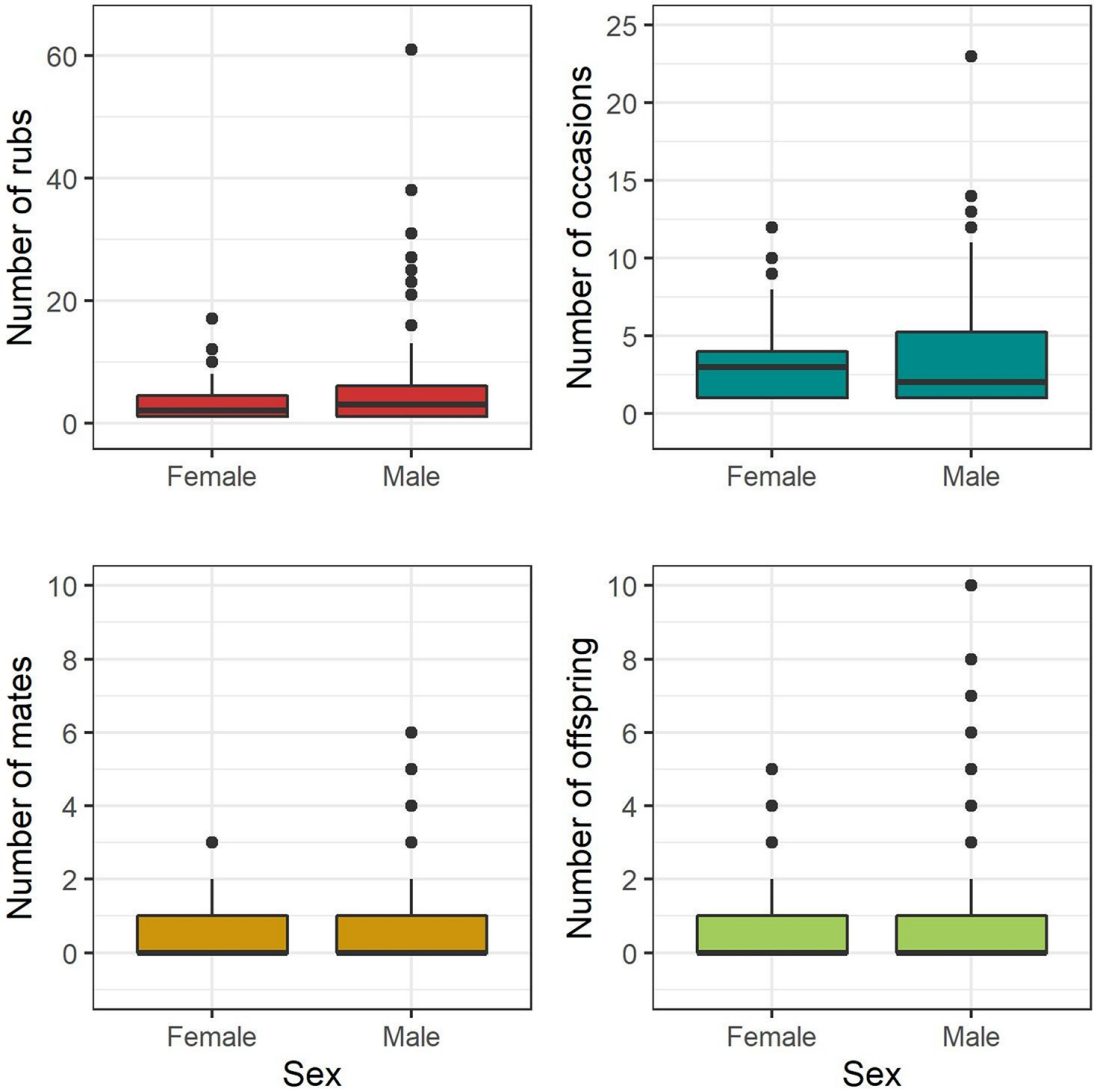
Summary plot. Summary plot of the number of rub objects at which and sampling occasions during which male and female brown bears were detected. Also displayed are the number of mates and offspring detected for each sex. Data are from southwestern Alberta 2011–2014.

Males were detected at a greater number of rub objects than females and during a more variable number of sampling occasions (Fig 2). The number of detected offspring ranged from 1–5 for females and 1–10 for males (Fig 2).

**Fig 3:**
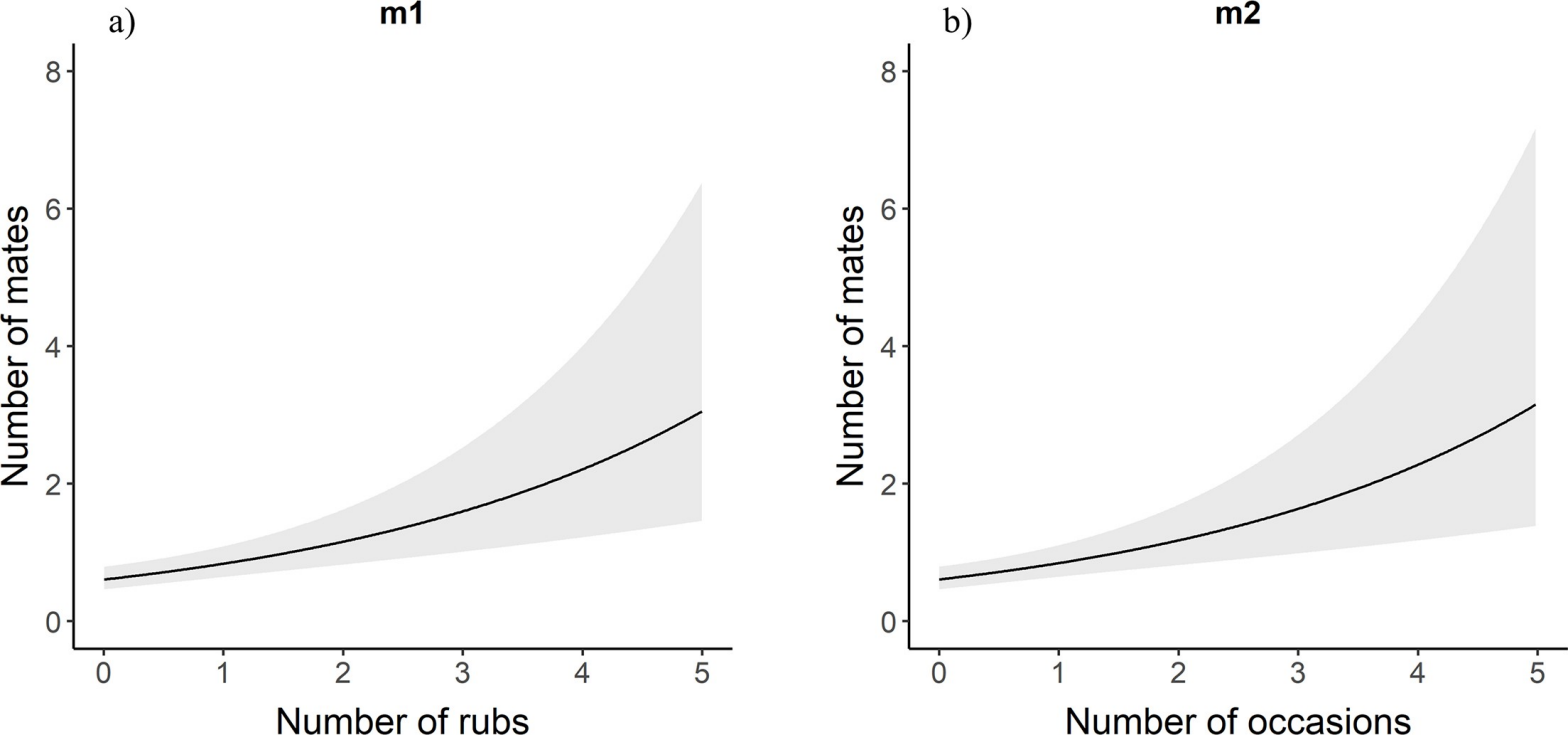
m1 and m2. Poisson regression response curves showing the relationship between the number of mates a male brown bear had and the number of rub objects (a) at which and sampling occasions (b) during which it was detected. 95% confidence intervals are in grey. Data are from southwestern Alberta 2011–2014.

**Fig 4:**
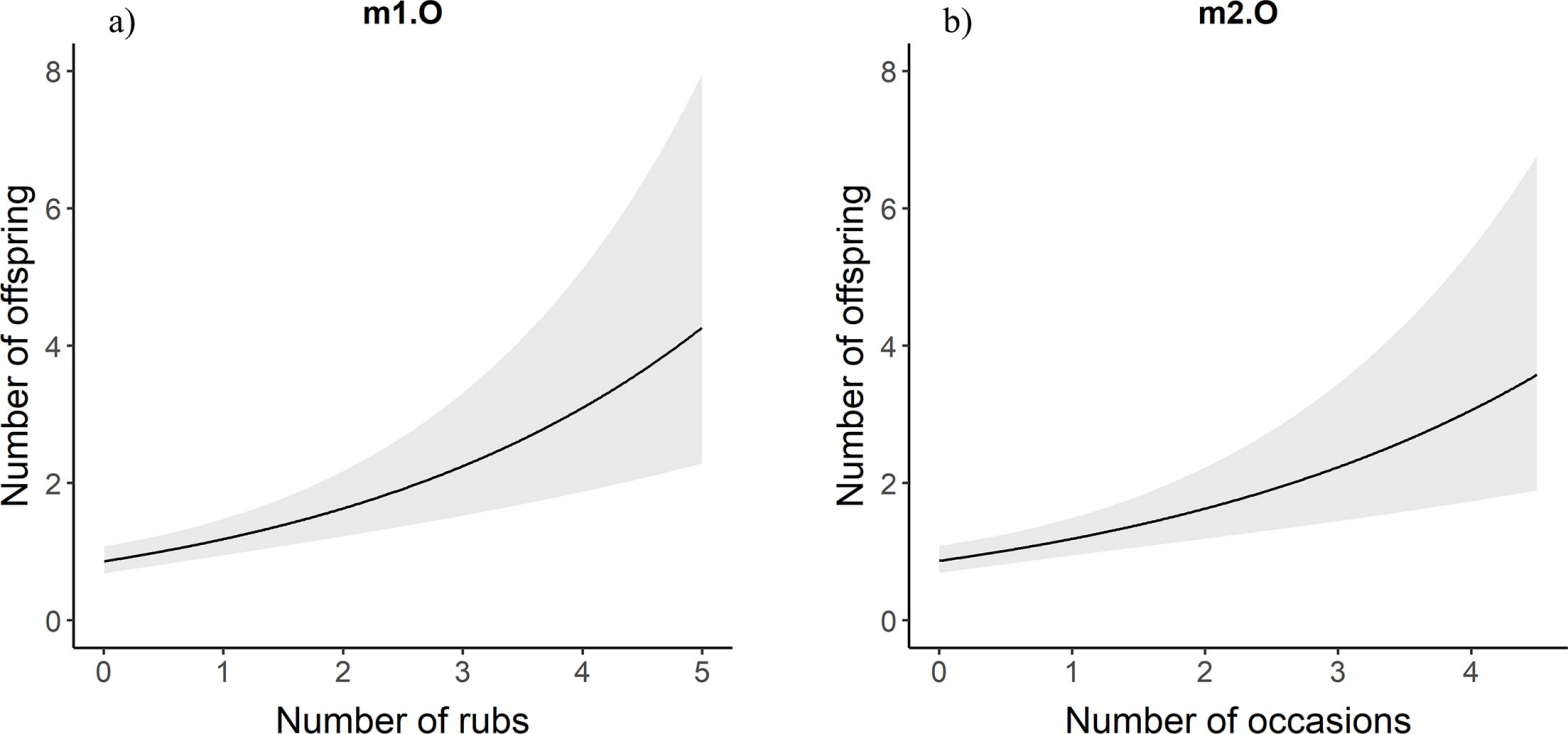
m1.O and m2.O. Poisson regression response curves showing the relationship between the number of offspring a male brown bear had and the number of rub objects (a) at which and sampling occasions (b) during which it was detected. Confidence intervals are in grey. Data are from southwestern Alberta 2011–2014.

**Fig 5:**
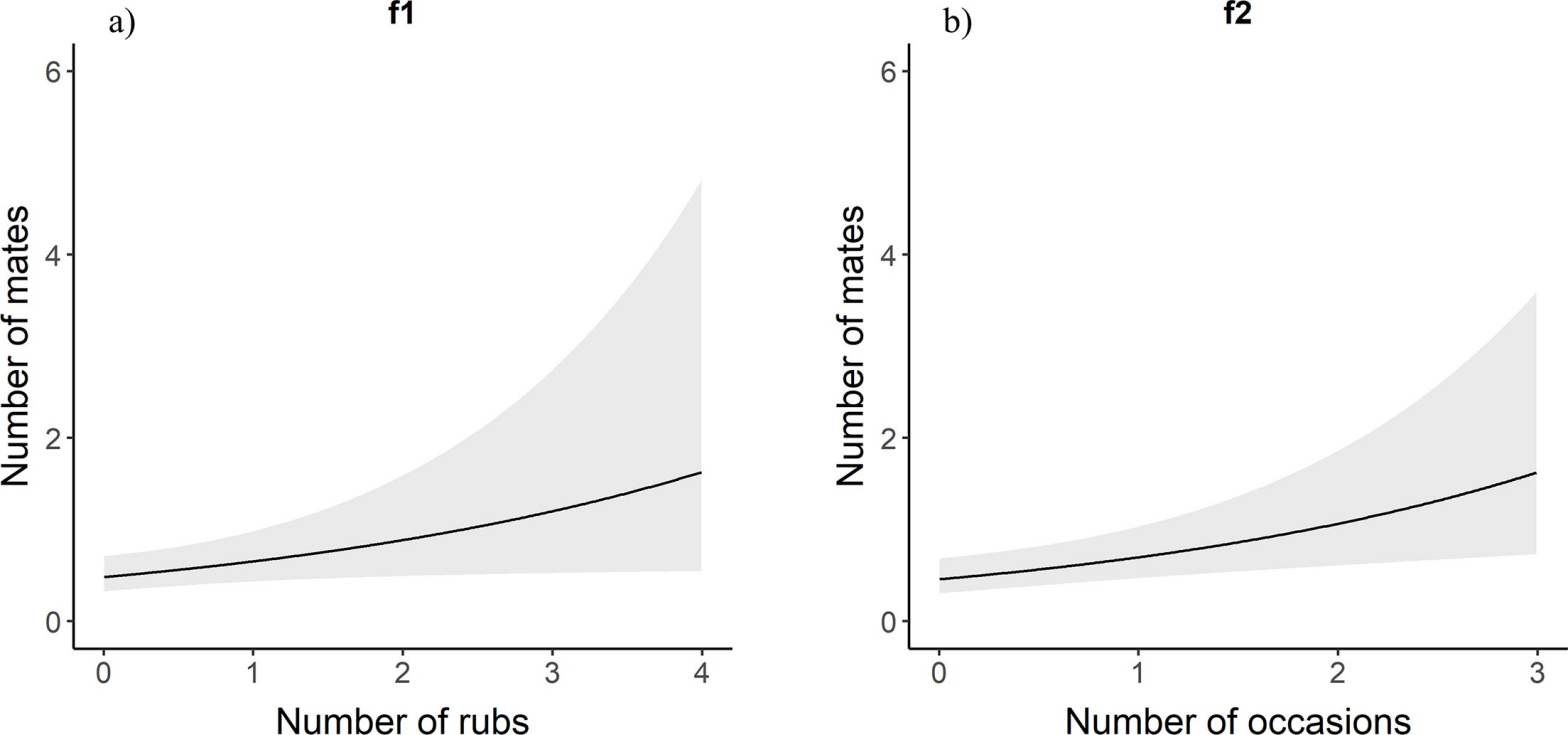
f1 and f2. Poisson regression response curves showing the relationship between the number of mates a female brown bear had and the number of rubbed objects (a) at which and sampling occasions (b) during which it was detected. Confidence intervals are in grey. Data are from southwestern Alberta 2011–2014.

**Fig 6:**
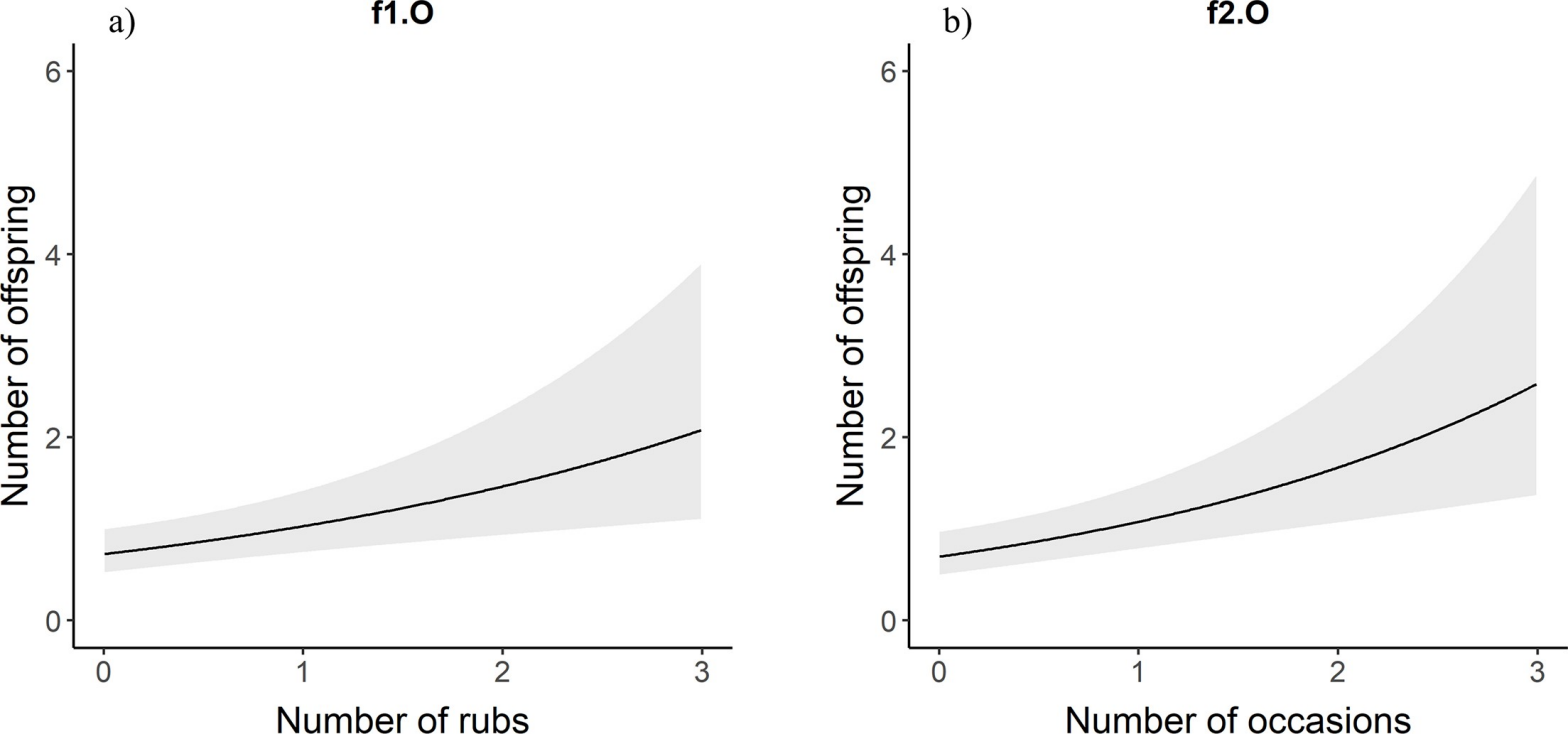
f1.O and f2.O. Poisson regression response curves showing the relationship between the number of offspring a female brown bear had and the number of rubbed objects (a) at which and sampling occasions (b) during which it was detected. Confidence intervals are in grey. Data are from southwestern Alberta 2011–2014.

For males in the full data set, the number of mates for a bear increased with the number of rub objects at which an individual was detected (Table 1, Fig 3). For each one unit increase in the number of rub objects at which a male bear was detected, the predicted number of mates increased by 1.38 times (Table 2). Similarly, male brown bears with more mates were detected in more occasions (Table 1, Fig 3). Likewise, males that had sired a greater number of offspring were detected at a greater number of rub objects and in more occasions (Table 1, Fig 4). For each additional occasion during which a male bear was detected, the predicted number of offspring is multiplied by 1.37 (Table 2). We observed the same relationships for female brown bears. Females with more mates were detected at more rub objects and in more occasions than females with fewer mates (Table 1, Fig 5). Likewise, there was a positive relationship between the number of offspring a female brown bear had and the number of rub objects and occasions that bear was detected at and in (Table 1, Fig 6). For each additional rub object at which and occasion during which a female was detected, the predicted number of offspring increased by 1.42 and 1.55 times respectively (Table 2).

**Table 1:** Poisson regression model results for the relationship between mates, offspring, and the number of rub objects at which and sampling occasions during which male and female brown bears were detected. Data are from southwestern Alberta 2011–2014.

				Full Data			Reduced Data—only bears known to have reproduced		
Sex	Model	Response Variable	Predictor Variable	Estimate	SE	*p*	Estimate	SE	*p*
Male	m1	number of mates	number of rubs	0.323	0.080	<0.001	0.193	0.105	0.065
			relative age				-0.040	0.142	0.778
	m2	number of mates	number of occasions	0.330	0.090	<0.001	0.194	0.106	0.066
			relative age				-0.034	0.140	0.808
	m1.O	number of offspring	number of rubs	0.320	0.070	<0.001	0.180	0.088	0.040
			relative age				0.029	0.111	0.793
	m2.O	number of offspring	number of occasions	0.315	0.080	<0.001	0.169	0.090	0.060
			relative age				0.035	0.109	0.745
Female	f1	number of mates	number of rubs	0.305	0.145	0.035	0.000	0.202	1.000
			relative age				0.200	0.178	0.263
	f2	number of mates	number of occasions	0.423	0.147	0.004	0.066	0.197	0.738
			relative age				0.183	0.185	0.323
	f1.O	number of offspring	number of rubs	0.352	0.113	0.002	0.071	0.158	0.652
			relative age				0.274	0.141	0.052
	f2.O	number of offspring	number of occasions	0.438	0.118	<0.001	0.063	0.160	0.693
			relative age				0.257	0.147	0.080

**Table 2:** Effect sizes for predictor variables of Poisson regression models. Data are from southwestern Alberta 2011–2014.

			Full Data		Reduced Data—only bears known to have reproduced	
Sex	Model	Predictor Variable	Exponential Effect Size	95% CI	Exponential Effect Size	95% CI
Male	m1	number of rubs	1.382	1.184–1.62	1.213	0.986–1.486
		relative age			0.961	0.728–1.270
	m2	number of occasions	1.391	1.164–1.668	1.214	0.989–1.500
		relative age			0.966	0.735–1.256
	m1.O	number of rubs	1.378	1.205–1.564	1.197	1.019–1.423
		relative age			1.029	0.835–1.271
	m2.O	number of occasions	1.371	1.178–1.590	1.184	1.003–1414
		relative age			1.036	0.841–1.281
Female	f1	number of rubs	1.356	1.024–1.795	1.000	0.676–1.465
		relative age			1.221	0.866–1.758
	f2	number of occasions	1.526	1.154–2.038	1.068	0.726–1.613
		relative age			1.201	0.849–1.713
	f1.O	number of rubs	1.422	1.136–1.785	1.074	0.803–1.463
		relative age			1.316	0.998–1.728
	f2.O	number of occasions	1.549	1.246–1.937	1.065	0.792–1.439
		relative age			1.294	0.977–1.749

Analysis of the reduced data set including only bears that were known to have successfully reproduced (*n* = 19 for females, *n* = 27 for males) led to reduced statistical significance in our hypothesized relationships. The confidence intervals of the relative age covariate overlapped zero in several models, and the relative age covariate was not significant in any model (Table 1). The positive relationship between the number of mates or offspring a bear had and the number of rub objects and number of occasions was consistent with the full data models (with the exception of model f1), but the only significant relationship was observed in model m1.O (Table 1). Similarly, effect size was lower for these models in all models for both males and females (Table 1).

## Discussion

Bears with a greater number of mates and a greater number of offspring were detected at more rub objects and during more occasions. Thus, our data supported our prediction of a positive relationship between bear rubbing behavior and reproductive success. Our results allow us to rule out hair removal as the sole motivation for rubbing because if this were the case, we would not expect a relationship between bear rubbing and reproductive success. Nevertheless, hair removal still could be a component of rubbing behavior. Although we cannot differentiate between rubbing for mate signaling and rubbing for dominance, both likely play a role in bear rub behavior. Detections of male brown bears at rub objects are typically highest during the breeding season [23], suggesting that male bears rub to signal for mates. However, as Lamb et al. [23] further hypothesize, bears might rub throughout the year to establish and maintain dominance hierarchies. Because bears rub throughout the active season [15], we can rule out mate signaling as the sole reason for rubbing behavior.

Rubbing for mate signaling can result in increased mating opportunities, higher-quality mates, and ultimately increased reproductive success and fitness. And for females, securing multiple mates might reduce the potential of SSI [35, 49]. Female promiscuity in mammals is a counterstrategy to SSI [62–64] because by mating with multiple males, the female can confuse paternity of her offspring and potentially reduce predation by infanticidal males [49]. Multiple-male mating has been observed in over 130 mammalian species, and females of some species will actively solicit copulations from multiple males [64]. Our results indicate that females that had successfully reproduced were detected at more rub objects than females without offspring. Thus, the mechanism behind rubbing behavior in females might go beyond mate advertisement; actively soliciting multiple male matings might confer fitness benefits to the rubbing female. Further, females that rub beyond the mating season might be relaying information on their quality as a mate that might inform future mating possibilities (e.g., whether reproduction was successful). Contrary to Clapham et al.’s [11] conclusion that females do not gain fitness benefits from rubbing, our results indicate that females with offspring were detected at a greater number of rub objects and during more occasions than females without offspring, suggesting there might be an individual fitness benefit to rubbing by females.

Female bears are induced ovulators (i.e., ovulation occurs after hormonal, physical, or behavioral stimulation), and multiple paternity of offspring in the same litter is possible [25, 32, 49, 65, 66]. For example, Shimozuru et al. [66] found that 14.6–17.1% of all brown bear litters evaluated were sired by multiple males. Thus, after mating, females might have the opportunity to choose among sperm of different males (cryptic female choice). Male-male competition, however, also can occur during this post-copulatory time via sperm competition [32, 44, 67]. Thus, if multiple mating reduces SSI by confusing paternity and females rub to attract multiple mates, this might explain the positive relationship between female reproductive success and rubbing.

Indeed, polygamous females can be problematic for males because copulation does not assure paternity. Thus, male brown bears are faced with a choice–guard the mated female for the duration of her oestrus, thereby assuring paternity [68, 69] but losing other mating opportunities, or solicit more copulations and potentially sire more offspring, but leave paternity to cryptic female choice and/or sperm competition. This decision might depend on the availability of breeding female bears [70]. When breeding females are scarce, it is likely in the males’ best interest to mate guard, but when breeding females are common, seeking additional copulations could be advantageous [70, 71].

We acknowledge that a limitation of our non-invasive hair sampling data is that we do not know the age of the bears in our analysis. Thus, we were not able to determine if a bear with no mates or offspring is because that bear was not successful in securing mates or because the bear was not of reproductive age. To address this as best as possible, we calculated a relative age covariate and included this in our secondary analysis. Datasets with relative age had less power because of lower sample sizes and thus, fewer degrees of freedom. The relative age covariate was not significant in any model and, for males, the same relationships between rubbing, mates, and offspring persisted. Further, previous research has found that subadults mark less frequently than adult brown bears [20, 72], and the function of scent marking by young bears remains unclear [11]. Because young bears rub less frequently than adults, it is unlikely that very many zeros in our data set are from bears of non-reproductive age.

Spatial clustering of rub objects also might play a role in our observed results. The ideal free distribution [73] predicts that higher quality bear habitat should have a higher density of bears. In turn, if bears rubbed more in higher quality areas, this spatial clustering of rub objects in high-quality habitats could be the ultimate cause of the relationship we found between rubbing and reproductive success. This line of reasoning implies a positive relationship between rubbing and bear density. However, recent work in the U.S. portion of this population did not find a consistent relationship between annual rub tree catch per unit effort and increasing density [30, 74]. Indeed, Lamb et al. [23] hypothesized the opposite–rubbing might be inversely related to population density. While local habitat conditions could influence use of rub trees [e.g., 30], the lack of a consistent pattern with density suggests that spatial clustering of rub objects is not the primary driver of our observed patterns.

In summary, our data suggest a fitness component to rubbing behavior. We conclude by proposing a new alternative hypothesis for consideration: female brown bears use the information obtained from olfactory cues of rubbing males throughout the season to choose offspring paternity. Data to examine this hypothesis are beyond the scope of our study but, if supported, this hypothesis would help explain the relationship between reproductive success and brown bear rubbing behavior. Because brown bears have delayed implantation and multiple paternity of offspring in the same litter is possible [25, 32, 49, 65, 66], females might be able to choose among sperm of different males. If so, female brown bears must rely on cues to determine which of the males that she has mated with will sire her offspring. Females might obtain this information from the olfactory and chemical signals deposited by rubbing males throughout the active season. These results indicate that rubbing is an adaptive behaviour in brown bears.

## Supporting information

S1 Data Poisson regression file for full and reduced data set. (XLSX)
